# Analysis of Airborne Fungal Communities on Pedestrian Bridges in Urban Environments

**DOI:** 10.3390/microorganisms11082097

**Published:** 2023-08-16

**Authors:** Amran A. Q. A. Al-Shaarani, Ziwei M. Quach, Xiao Wang, Mohammed H. M. Muafa, Md M. H. Nafis, Lorenzo Pecoraro

**Affiliations:** School of Pharmaceutical Science and Technology, Tianjin University, 92 Weijin Road, Tianjin 300072, China; amran303@163.com (A.A.Q.A.A.-S.); ziweimelaniequach@tju.edu.cn (Z.M.Q.); wang_xiao1996@163.com (X.W.); muafa99@tju.edu.cn (M.H.M.M.); mh.nafis29oct@gmail.com (M.M.H.N.)

**Keywords:** airborne fungi, concentration, distribution, diversity, environmental factors, fungal community structure, outdoor environments

## Abstract

Airborne fungal spores constitute an important type of bioaerosol and are responsible for a number of negative effects on human health, including respiratory diseases and allergies. We investigated the diversity and concentration of culturable airborne fungi on pedestrian bridges in Tianjin, China, using an HAS-100B air sampler. We compared the airborne fungal communities at the top central area of the selected pedestrian bridges and along the corresponding sidewalk, at ground level. A total of 228 fungal strains belonging to 96 species and 58 genera of Ascomycota (68.86%), Basidiomycota (30.26%), and Mucoromycota (0.88%) were isolated and identified using morphological and molecular analysis. *Alternaria* was the dominant genus (20.61%), followed by *Cladosporium* (11.48%), *Schizophyllum* (6.14%), *Sporobolomyces* (5.70%), and *Sporidiobolus* (4.82%). *Alternaria alternata* was the most frequently occurring fungal species (6.58%), followed by *Schizophyllum commune* (5.26%), *Alternaria* sp. (4.82%), *Sporobolomyces carnicolor* (4.39%), and *Cladosporium cladosporioides* (3.95%). The recorded fungal concentration ranged from 10 to 180 CFU/m^3^. Although there was no significant difference in the distribution and abundance of the dominant airborne fungal taxa between the two investigated bridges’ sites, numerous species detected with a low percentage of abundance belonging to well-known pathogenic fungal genera, including *Alternaria*, *Aspergillus*, *Aureobasidium*, *Cladosporium*, *Penicillium*, and *Trichoderma*, were exclusively present in one of the two sites. The relative humidity showed a stronger influence compared to the temperature on the diversity and concentration of airborne fungi in the investigated sites. Our results may provide valuable information for air quality monitoring and for assessing human health risks associated with microbial pollution.

## 1. Introduction

Fungi are among the most abundant organisms in the biosphere, playing a number of very important roles in the environment. For instance, fungi act as decomposers, breaking down dead organic matter and replenishing the soil with essential nutrients. They also provide numerous benefits to modern society through their use in the pharmaceutical, beverage, and food industries [1,2,3,4]. However, fungi are also considered a source of air pollution, causing various diseases and allergies, and being responsible for a number of infections in humans and animals [5,6]. Fungal spores constitute an important type of bioaerosol and can be found in far higher numbers than pollen grains in the air [7]. Numerous previous studies have reported a strong connection between many adverse health problems and long-term exposure to fungal spores [8,9,10,11,12]. Over 80 fungal species have been identified as the source of respiratory tract allergy symptoms, and more than 100 fungal genera have been reported to be associated with human and animal severe infections [7]. Infectious diseases resulting from the inhalation of different fungi are determined by the quantity and size of inhaled fungal particles. Fungal spores with a diameter of less than 5 µm are capable of penetrating the alveoli, resulting in allergic alveolitis and other serious diseases [7,13]. Spores of airborne fungi can cause type I hypersensitivity and they can also induce some respiratory reactions [7]. Previous studies have found that airborne fungi can be linked to human lung cancer when the fungus *Aspergillus flavus*, which produces aflatoxin B, enters the human respiratory tract via inhalation or ingestion [14,15,16,17].

Many studies have found that environmental factors, such as temperature, humidity, sunlight intensity, human activity, and pollution have an impact on the concentration, diversity, and survival of airborne fungi [18,19]. *Cladosporium*, *Alternaria*, *Penicillium*, and *Aspergillus* have been found to be the dominant airborne fungal genera in various outdoor environments and their concentrations have been shown to vary under the influence of different environmental parameters [12,18]. Exposure to the aforementioned fungi has been found to be responsible for some respiratory diseases such as asthma and allergic rhinitis [20,21,22]. A considerable number of studies have been conducted in different Chinese cities, focusing on both indoor and outdoor airborne fungal communities [6,23,24,25,26,27,28]. These studies have provided valuable information related to the diversity of airborne fungal communities in the analyzed environments, which certainly can be used to understand implications for human health, for risk assessment, and for prevention of fungus-associated diseases [24,29]. However, there is still much to be learned about the composition, structure, and dynamics of fungal communities in urban areas, which are characterized by the presence of an enormous number of indoor and outdoor air environments offering a variety of conditions for the presence of different fungi. A substantial portion of these urban environments is nearly completely unexplored.

Pedestrian bridges serve as public spaces for urban mobility [30]. They are designed to be built over streets to allow an easy flow of people without impeding vehicular traffic movement to avoid traffic jams [31,32]. However, while walking on pedestrian bridges, people could be exposed to a variety of potentially harmful fungal particles suspended in the air by the vehicles moving along the underlying road. In addition, the presence of pedestrian bridges enables drivers to increase their vehicle speed, causing an intensification of car exhaust emissions and air pollution, which in turn may affect the concentration of airborne fungi in such places [33,34,35]. Air pollution not only harms human health by causing pathological conditions, allergies, and other health problems, but also has a significant negative impact on the socioeconomic development process in most of China’s northern cities [36].

Our study, carried out in Tianjin, China, aimed to assess the diversity and concentration of airborne fungi on pedestrian bridges and to understand the effect of various environmental factors on the analyzed fungal community structure. We compared the airborne fungal communities at the center of the selected pedestrian bridges with the communities found at ground level, along the corresponding sidewalk, in order to test our hypothesis of a particularly high diversity and/or concentration of airborne fungi expected on the bridges, which could have implications for human health. Since many people use pedestrian bridges on a daily basis, it is of critical importance to study the airborne microorganisms in these areas in order to assess health risks, and provide valuable information for air quality monitoring and microbial pollution control.

## 2. Materials and Methods

### 2.1. Sampling Locations

The present study was conducted in Tianjin, China’s fifth largest city, located in the northeastern part of China, 135 km southeast of Beijing. In terms of number of inhabitants, Tianjin has the fourth largest urban population in China with 13,794,450 people in the year 2021 (data retrieved from https://worldpopulationreview.com/world-cities/tianjin-population, accessed on 7 April 2022 and https://www.nationsonline.org/oneworld/map/google_map_Tianjin.htm, accessed on 23 April 2022).

Ten sampling sites located on five different pedestrian bridges were selected for this study. For each bridge, two sites were analyzed, the top central area of the bridge (C sites) and the ground level area on the sidewalk under the bridge (U sites; Supplementary Table S1 and Figure S1).

### 2.2. Sampling Method

At each experimental location, sampling was conducted three times: once a week for three consecutive weeks from 13 to 28 August 2021. Air samples were collected when the weather was dry and stable, approximately between 8:00 and 10:00 o’clock. An air sampler HAS-100B (Hengao T&D, Beijing, China) was employed to collect the culturable airborne fungi from each studied site using sterile petri dishes of 9 cm in diameter, containing Malt Extract Agar (MEA) supplemented with chloramphenicol (100 mg/L) to inhibit bacterial proliferation. The sampler was fixed on a stand at about 1.5 m above ground level. Air samples were collected at an air flow of 100 L/min, with a rotating dish speed of 0–4 rpm, in a 10 min sampling operation per site. In order to avoid cross-contamination, the air sampler was sterilized before each sampling procedure by swabbing every surface with cotton dipped in 70% ethanol. During the sampling process, environmental parameters including temperature and relative humidity were measured at each sampling location using a TES 1364 Humidity-Temperature Meter (Taiwan, China). The collected samples were taken to the lab, where the cultured petri dishes were incubated at room temperature for 5–7 days in the darkness. The total number of growing fungal colonies was counted, and each strain was picked up and inoculated into a new petri dish to obtain pure cultures (Supplementary Figure S2). All isolated fungal strains were deposited in the LP Culture Collection (personal culture collection held in the laboratory of Prof. Lorenzo Pecoraro) at the School of Pharmaceutical Science and Technology, Tianjin University, Tianjin, China.

### 2.3. Enumeration of Fungi

Fungal colonies were enumerated after incubation and the samples’ concentrations were expressed as Colony-Forming Units per cubic meter of air (CFU m^−3^) using the number of isolated fungi at each sampling site according to the formula below:

$$N=\frac{Cn*1000}{t*V}$$

where *N* = concentration of fungal colonies in CFU/m^3^, *Cn* = number of fungal colonies, 1000 = conversion factor of liter to cubic meter, *t* = sampling operation time, and *V* = velocity of the air flow.

### 2.4. Fungal Identification

Identification of all isolated fungal strains was performed using DNA-based methodology combined with microscopy. The DNA was extracted using the cetyltrimethylammonium bromide method (CTAB) [37,38].

Fungal rRNA gene internal transcribed spacer (ITS) region was amplified using the universal primer pair ITS1 (5′-TCCGTAGGTGAACCTGCGG-3′)/ITS4 (5′-TCCTCCGCTTATTGATATGC-3′) according to White et al. [39]. The reaction mixture for PCR (50 µL) consisted of 25.0 µL of 2 × Rapid Taq Master Mix (Vazyme Biotech Co. Ltd., Nanjing, China), 2 µL of forward primer (10 µM), 2 µL of reverse primer (10 µM), 2.0 µL (20 ng DNA) of template, and 19 µL of double distilled sterilized water. The PCR amplifications were performed by set-up program: initial denaturation at 98 °C for 3 min, 30–35 cycles of 98 °C for 10 s, annealing at 55 °C for 15 s, extension at 72 °C for 15 s, and final extension at 72 °C for 2 min. Gel electrophoresis was used for the detection of the PCR products using an electrophoresis tank (LiuYi, Beijing, China) on a 1% agarose gel. Sequencing of the PCR products was performed at Tsingke Biological Technology Company (Beijing, China). The obtained DNA sequences were analyzed with the Basic Local Alignment Search Tool (BLAST) program of NCBI (the National Center for Biotechnology Information, Bethesda, MD, USA, http://www.ncbi.nlm.nih.gov/Blast.cgi, accessed on 21 March 2023) in order to determine the best matches that enabled taxonomic identification. DNA sequences were deposited in GenBank (Accession Nos. ON705346–ON705573). Macroscopic and microscopic observations were performed to characterize fungal morphology [40,41]. A Nikon ECLIPSE Ci-L microscope was used to examine fungal morphological characters including branched septate hyphae, conidiophores, conidia, arthroconidia, poroconidia, etc.

### 2.5. Statistical Analysis

Fungal diversity was evaluated using Shannon and Chao1 indexes. All subsequent analyses were conducted using R (version 4.2.2 R; [42]). Kruskal–Wallis tests [43] were used to compare variations of Shannon and Chao index among multiple groups, while pairwise comparisons were performed by Wilcoxon rank-sum test [44]. Distance-based redundancy analysis (dbRDA) was performed based on Bray–Curtis distance matrix using vegan package (version 2.6-4; [45]). Statistical significance of environmental factors was evaluated by permutation test (permutations = 999). Correlations between fungal genera and environmental parameters were evaluated based on Spearman’s correlation coefficient (r) and visualized by pheatmap (version 1.0.12; [46]). Between-group Venn diagrams were plotted to identify unique and common fungal genera. Principal co-ordinates analysis (PCoA) and analysis of similarity (ANOSIM) with 999 permutations were used to compare dissimilarities between samples in different locations based on the Bray–Curtis distance [47] in R package.

## 3. Results

**Figure 1:**
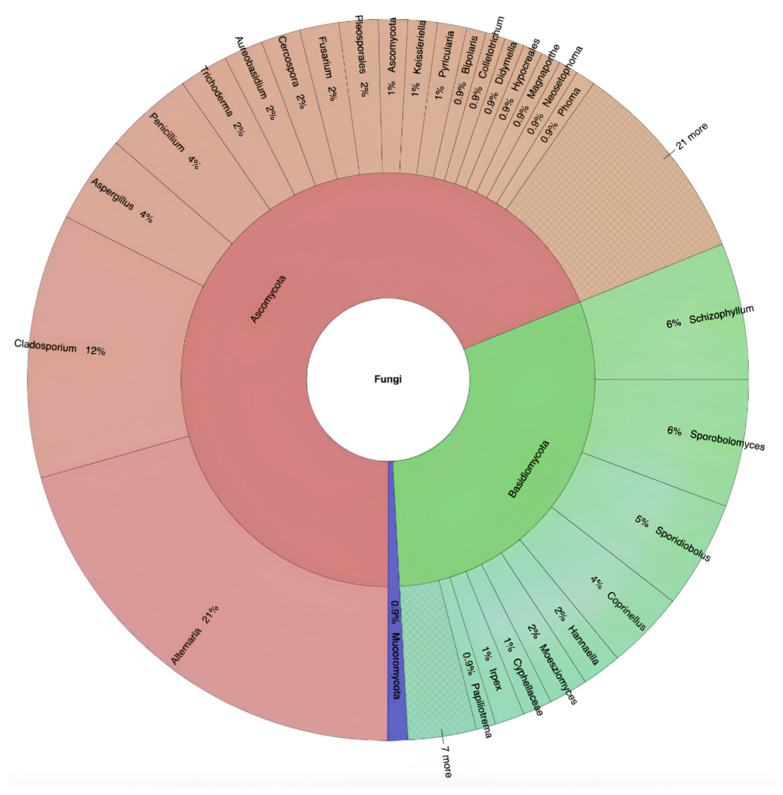
Taxonomic identification and relative abundance of airborne fungi isolated from all selected pedestrian bridges.

A total of 228 fungal strains belonging to 96 species and 58 genera of Ascomycota (68.86%), Basidiomycota (30.26%), and Mucoromycota (0.88%) were isolated in three consecutive weeks of sampling from 10 sites located on five different bridges (Figure 1 and Supplementary Table S2).

At genus level, we found that *Alternaria* and *Cladosporium* were the most diverse taxa, each including nine identified species, which accounted for 9.38% of all the isolated airborne fungal diversity. The genus *Aspergillus*, with six species (6.25%), was the second most diverse taxon, followed by *Cercospora*, with four species (4.17%), and the three genera *Sporidiobolus*, *Penicillium*, and *Trichoderma*, each with three species (3.13%). Looking at the contribution of each sampling site to the total fungal species diversity observed in this study, BJD (U) and WJY (U) yielded the highest percentage of species (14%), followed by NMWA (C) and NG (C) (12%), BJD (C) (9%), WJY (C), WJR (U), WJR (C), and NG (U) (8%), with the lowest species diversity recorded from NMWA (U) (7%).

**Figure 2:**
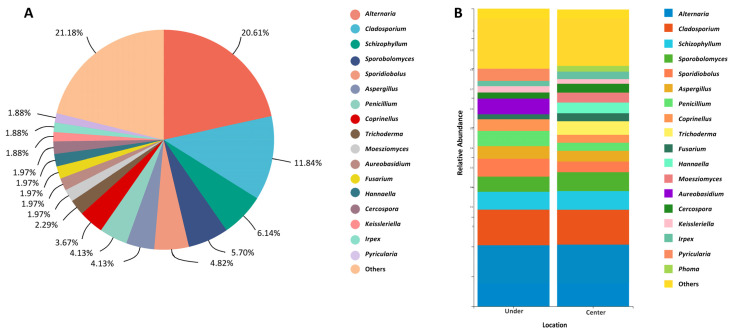
Relative abundance of airborne fungal genera isolated from the analyzed bridges. (**A**) Overview of total fungal diversity from all sampling sites. (**B**) Genus-level fungal diversity at the center of and under the bridges. Fungal genera with very low relative abundances (<1%) were merged as “others” in the bar plot.

Considering the number of isolated strains, the five most abundant fungal genera were *Alternaria* (47 strains, 20.61%), *Cladosporium* (27 strains, 11.84%), *Schizophyllum* (14 strains, 6.14%), *Sporobolymyces* (13 strains, 5.70%), and *Sporidiobolus* (11 strains, 4.82%) (Figure 2A, Supplementary Table S3). Looking separately at the different sampling locations, from the sites under the bridges, six fungal genera, *Alternaria* (20.51%), *Cladosporium* (11.97%), *Schizophyllum* (5.98%), *Sporidiobolus* (5.98%), *Sporobolomyces* (5.13%), and *Penicillium* (5.13%), showed a strain relative abundance above 5%, whereas, from the sites at the center of the bridges, the number of genera showing a relative abundance higher than 5% was four, including *Alternaria* (20.72%), *Cladosporium* (11.71%), *Schizophyllum* (6.31%), and *Sporobolomyces* (6.31%), with the three dominant genera *Alternaria* (20.51–20.72%), *Cladosporium* (11.97–11.71%), and *Schizophyllum* (5.98–6.31%) keeping a very stable relative abundance at both locations (Figure 2B). Overall, 51% of the fungal strains were collected from sidewalks under the studied bridges (U sites), while 49% were collected from the center of the bridges (C sites).

**Figure 3:**
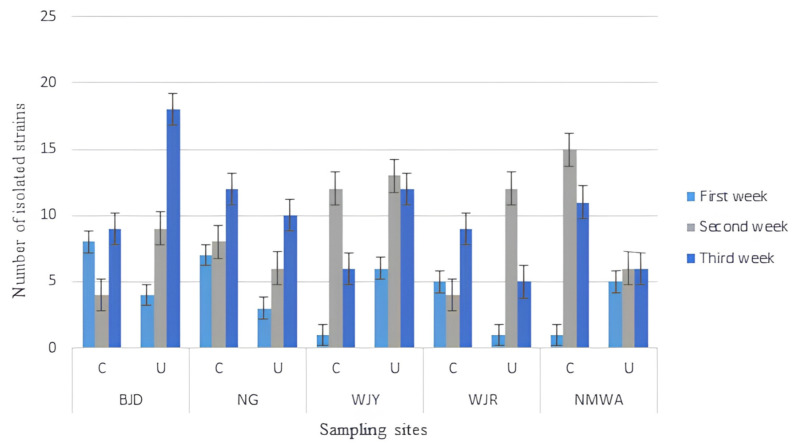
Total isolated fungal strains from each sampling week, site, and location (C = the top central area of the bridges; U = under the bridges along the side walk). Error bars represent SEM.

**Figure 4:**
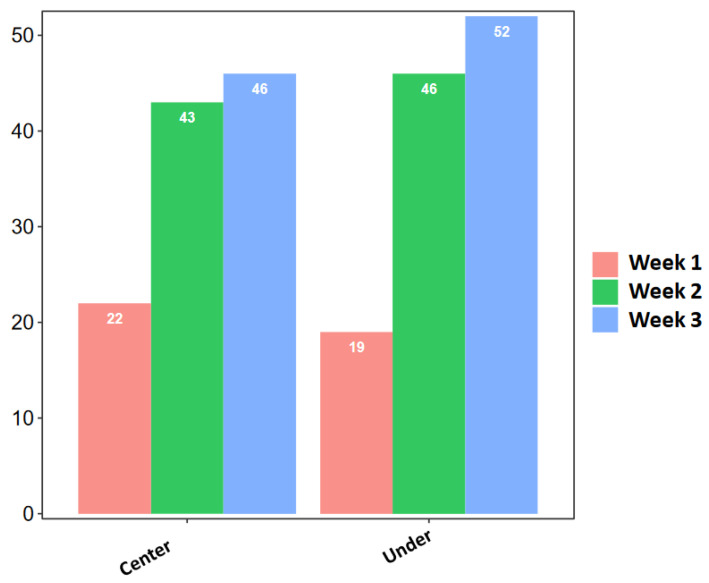
Total isolated strains in the 3 sampling weeks from the center of and under the analyzed bridges.

Looking at the different sites and weeks of sampling (Figure 3), the site BJD (C) showed the highest fungal diversity during the first week (8 strains), yielding 19.5% of the total collected strains from the analyzed bridges. In week 2, the highest number of strains (15) was isolated from the site NMWA (C), accounting for 16.9% of the total recorded fungi. During week 3, BJD (U) yielded the highest number of fungal strains (18), representing 18.4% of the total strains collected. Overall, the third week of sampling showed the highest fungal presence, yielding a total number of 98 strains, 46 strains collected at the center of the analyzed bridges and 52 collected under the bridges, followed by the second week with 89 strains. The first week showed the lowest presence of fungi (41 strains), with 22 and 19 strains collected at the center of and under the bridges, respectively (Figure 4).

*Alternaria* was the most abundant fungal genus found across the three different sampling weeks, with the highest percentage (78.72%) recorded in the third week. All the *Schizophyllum* strains isolated in this study were collected in the second sampling week, when 92.31% of *Sporobolomyces* strains were also isolated, together with the great majority of strains belonging to the genera *Sporidiobolus* (90.91%) and *Coprinellus* (87.50%). The highest relative strain abundance (66.67%) of both *Aspergillus* and *Penicillium* was found in the first and third week of sampling, respectively.

*Alternaria alternata* showed the highest relative abundance in the third week, *Schizophyllum commune* and *Sporobolomyces carnicolor* were only recorded in the second week, while *Alternaria* sp., *Cladosporium cladosporioides*, and *Penicillium oxalicum* were all found to be more common in the third week.

*Alternaria alternata* was the most abundant fungal species recorded in this study, with 15 strains, accounting for 6.58% of the total isolated strains, followed by *Schizophyllum commune* (12 strains, 5.26%), *Alternaria* sp. (11, 4.82%), *Sporobolomyces carnicolor* (10, 4.39%), *Cladosporium cladosporioides* (9, 3.95%), *Sporidiobolus pararoseus* (8, 3.51%), *Coprinellus radians* and *Penicillium oxalicum* (both with seven strains, 3.07%), and *Alternaria tenuissima* and *Cladosporium tenuissimum*, both yielding 2.63% of total strains.

The fungal concentration, expressed in CFU/m^3^, varied at each sampling location, site, and week (Supplementary Figure S3), ranging between 10 and 180 CFU/m^3^ (Supplementary Table S4). The sampling site BJD (U) showed the highest fungal concentration value during week 3 (180 CFU/m^3^), followed by NMWA (C) with 150 CFU/m^3^ at week 2, while the lowest concentration (10 CFU/m^3^) was measured at the three sampling sites WJY (C), WJR (U), and NMWA (C), during the first week of sampling. The highest average fungal concentration (103.33 CFU/m^3^) was recorded at BJD (U) and at WJY (U), whereas the lowest value (56.67 CFU/m^3^) was measured at NMWA (U) (Table 1 and Supplementary Figure S4).

The fungal concentration at the center of the bridges was constantly higher in week 3 than in week 1, whereas week 2 showed in some sites (NMWA and WJY) the highest concentration and in some other bridges the lowest (BJD and WJR; Supplementary Figure S4). For the sites under the bridges, the fungal concentration was relatively stable during the sampling period under the bridge NMWA, compare to the other sites, being 50 CFU/m^3^ at week 1, and slightly increasing to 60 CFU/m^3^ at weeks 2 and 3. During all 3 weeks, at site NG, the level of CFU/m^3^ under the bridge was always lower than the concentration at the center of the bridge. On the contrary, at the site WJY, the CFU concentration under the bridge was higher than the concentration at the center of the bridge during the whole sampling period (Supplementary Figure S4).

The two investigated locations (C and U) showed very similar CFU/m^3^ concentrations of the five most abundant fungal genera (*Alternaria*, *Cladosporium*, *Schizophyllum*, *Sporobolomyces*, and *Sporidiobolus*; Supplementary Figure S5).

**Figure 5:**
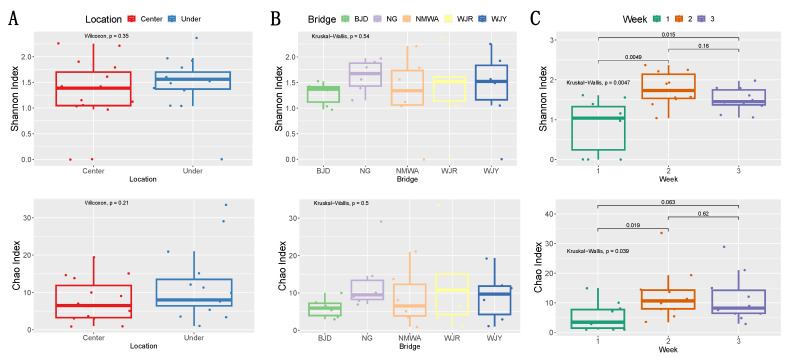
Fungal alpha diversity (Shannon and Chao indexes) in different locations at the center of and under the analyzed bridges (**A**) at different bridges (**B**) and sampling weeks (**C**). Differences between groups were evaluated by Wilcoxon rank-sum test and Kruskal–Wallis test as indicated by *p*-values.

Table 2 shows the Shannon index (H′) values for fungal diversity in different sampling locations for each sampling week. Airborne fungal diversity in C sites showed a peak at 2.21 in the second week and the lowest value (0) in the first week, while, for the U sites, the highest Shannon index value (2.37) was recorded in the second week and the lowest (0) in the first week. The alpha diversity of airborne fungal communities under the studied bridges was higher than at the center of the bridges (Figure 5A). Site NG showed the highest fungal diversity according to the Shannon index, while site WJR was characterized by the richest fungal community based on the Chao1 Index (Figure 5B). During week 2, both fungal richness and diversity reached the maximum level of the whole sampling period (Figure 5C).

**Figure 6:**
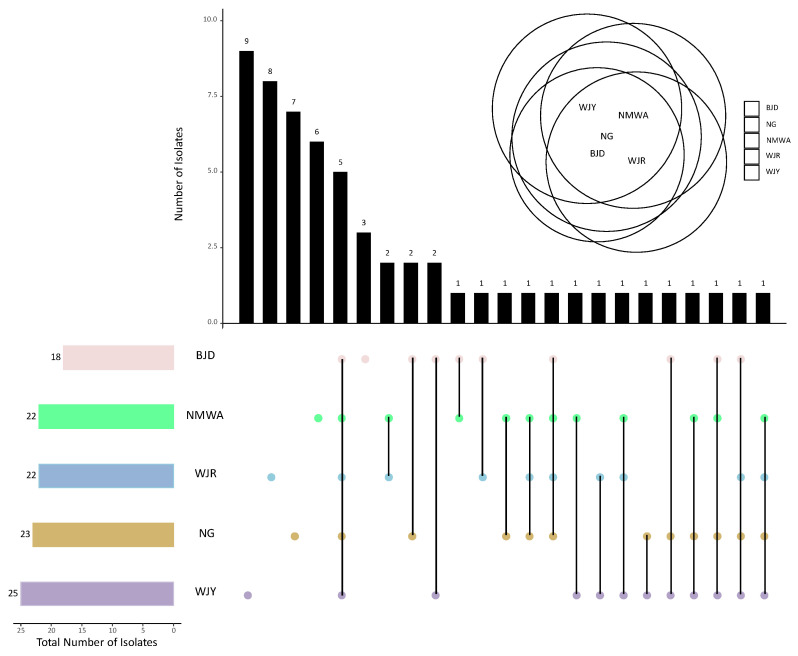
Number of common and unique fungal genera collected from the five sampling bridges by upset-Venn diagram (bridge names are abbreviated as described in the Supplementary Table S1).

Out of the 58 fungal genera found in the whole study, 22 were found to be present at both bridge sites (C and U), including the five dominant genera *Alternaria*, *Cladosporium*, *Schizophyllum*, *Sporobolomyces*, and *Sporidiobolus*, while 17 and 19 genera were unique to C sites and U sites, respectively (Supplementary Figure S6, Supplementary Table S5). The five studied bridges shared the five fungal genera *Alternaria*, *Cladosporium*, *Coprinellus*, *Sporobolomyces*, and *Schizophyllum*, while the genera *Aspergillus*, *Moesziomyces*, *Penicillium*, and *Sporidiobolus* were found in four out of five bridges. The highest total number of genera and the highest number of unique genera (nine) were recorded from bridge WJY (Figure 6). Bridge WJR showed eight exclusive genera, while for sites NG, NMWA, and BJD, seven, six, and three unique strains were reported, respectively (Figure 6).

The variations of fungal genera were analyzed by principal co-ordinate analysis (PCoA). The distributions of fungal communities under the bridges and at the center of the bridges were distinguished based on Bray–Curtis distance. The *r* value was not close to 1 (r = 0.0119), thus suggesting that the difference between groups was similar to the difference within groups. The *p* value calculated from ANOSIM was 0.441 (*p* > 0.05), indicating that differences in fungal communities between the two sites (at the center of and under the bridges) were not statistically significant. The latter result was confirmed by the Wilcoxon rank-sum test used to explore the variation of abundant genera among the two sites, which were not significantly differentiated. For instance, *Alternaria*, which was the dominant genus at both bridge sites, did not show any significant difference between the center of and under the analyzed bridges (*p* = 0.9258; Supplementary Figure S7).

**Figure 7:**
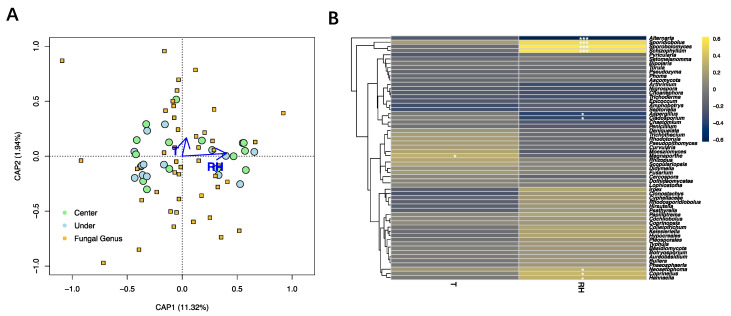
Influence of environmental variables on the fungal community. (**A**) Distance-based redundancy analysis of the fungal communities, with symbols coded by sampling locations and fungal genera. (**B**) Correlation heatmap between the detected fungal genera and environmental factors. Different colors refer to Spearman’s correlation coefficient (r). The *p* value is indicated by: * *p* < 0.05, *** *p* < 0.001. T = temperature, RH = relative humidity.

The dbRDA based on fungal genera showed that relative humidity was significantly correlated with fungal community composition (*p* = 0.001; Figure 7A, Supplementary Table S6). The influence of temperature on the analyzed fungal diversity was not significant (*p* = 0.413). Nine fungal genera were correlated to the relative humidity with *p*-values less than 0.05. More specifically, *Alternaria*, *Aspergillus*, and *Cladosporium* were negatively correlated with the latter environmental factor, while *Sporidiobolus*, *Coprinellus*, *Neosetophoma*, *Schizophyllum*, *Sporobolomyce*, and *Hannaella* were positively correlated. Only one fungal taxon (*Magnaporthe*) was positively correlated with the temperature of the sampling site (Figure 7B).

## 4. Discussion

This is the first study to assess the concentration and diversity of culturable airborne fungi on pedestrian bridges in Tianjin, China. The five analyzed pedestrian bridges, each comprising two sampling sites (C and U sites), showed a rich fungal community, which resulted in the isolation of 228 strains belonging to 96 species and 58 genera. Previous studies have shown that outdoor airborne fungal diversity and concentrations varied widely between cities in different regions, under the influence of various environmental factors, including traffic flow, human activities, and vegetation presence. Numerous studies on airborne fungal communities have been conducted in different Chinese cities [26,48]. For example, a study conducted at three different sampling sites in Beijing showed that the fungal concentration ranged from 4.8 × 10^2^ CFU/m^3^ to 2.4 × 10^4^ CFU/m^3^, whereas the concentration of culturable fungi was found to range from <12 to 8767 CFU/m^3^ in four sampling locations in Hangzhou [24].

*Alternaria*, *Cladosporium*, *Schizophyllum*, *Sporobolomyces*, and *Sporidiobolus* were the most frequently occurring fungal genera in the sampling sites analyzed in our study. Similarly, *Alternaria* and *Cladosporium* have been identified as the dominant airborne fungi in previous studies conducted in Beijing, Hangzhou, Nanjing, and Tianjin [24,26,28,29]. *Alternaria* was the most common fungal genus recorded in our study, accounting for 20.61% of the total isolated strains. This finding is consistent with a previous study conducted by our research group at city level in Tianjin, in which *Alternaria* was the most frequent genus, yielding roughly one-fourth of the total isolated airborne fungal strains [26]. The most frequent *Alternaria* species found in our analyzed environments was *A. alternata*, a widespread and common allergenic fungus that causes immunoglobulin E (IgE)-mediated respiratory diseases, particularly asthma exacerbation [49]. In addition, *A. alternata* has been detected as the most common airborne fungal species in nasal discharge, inducing strong immunologic activity in nasal epithelial cells and playing an important role in the pathogenesis of chronic rhinosinusitis (CRS) [50]. While *A. alternata* was present at very similar levels in both C and U sites of the analyzed bridges, several *Alternaria* species were exclusive to one of the two sites. For instance, *A. brassicae* and *A. tamaricis* were only isolated at the top central areas of bridges, whereas *A. porri* was exclusively reported in the sites under the bridges. Further studies are needed to clarify the possible effect on human health caused by the presence in the air of the numerous *Alternaria* species detected in our study sites, given that the whole genus *Alternaria* has been reported among the most common allergenic microorganisms [27,51,52]. *Cladosporium* was the second most abundant fungal genus recorded in the present study. This taxon was also previously reported as the second most common genus in Tianjin city’s outdoor environments by [26] and found to be dominant in the atmosphere of Beijing, China [29]. *Cladosporium* fungi reach their optimal growth at temperatures ranging from 18 to 28 °C in humid, wet environments [53,54,55]. Therefore, it is not surprising that *Cladosporium* fungi were found to be highly abundant in our study, where the recorded average temperature was 25.96 °C and the relative humidity was consistently high during the whole sampling period, with an average value of 81.03%. Additionally, *Cladosporium* species spores are known to be widely dispersed in the air worldwide and contribute to the larger portion of airborne spores in temperate climates [56]. *Cladosporium* fungi have been reported to be associated with human health problems, causing asthma and inducing allergies [55,57]. Although most *Cladosporium* species are not pathogenic; some of them may cause ear, eye, and nose infections. In a previous study conducted in Spain, based on the microbial analysis of 135 nasal samples from either healthy or allergic people, *Cladosporium* was the most frequently found genus [58]. Another study conducted in China reported the species *C. cladosporioides* to be a cause of phaeohyphomycotic dermatitis in giant pandas [59]. Given that *Cladosporium* fungi are responsible for numerous negative effects of human health, the exclusive presence of several *Cladosporium* species, including *C. cucumerinum*, *C. delicatulum*, *C. oryzae*, *C. sphaerospermum*, and *Cladosporium* sp., in one out of the two analyzed bridges’ sites (C and U sites) could represent a factor worthy of further studies in order to understand the potential effect on pedestrians’ health. The genus *Schizophyllum*, showing the third highest abundance in our analyzed environments, is known mostly as a saprotrophic genus, commonly growing on wood and fallen leaves [60,61]. *Schizophyllum commune,* which accounted for 5.26% of the total isolated strains in this study, was recently found to cause mycotic disease in people on a rare basis, especially via cutaneous infection [62]. Although this fungal species is not commonly considered as pathogenic to humans, being mostly regarded as a medicinal mushroom [63], *S. commune* was indicated as a possible cause of pulmonary basidiomycosis by Unno et al. [64], while inhalation of *S. commune* spores has been recently linked to allergic bronchopulmonary mycosis (ABPM) or allergy-related bronchopulmonary infections and sinusitis [65,66]. Furthermore, *S. commune* infection cases have increased significantly in the last few decades, since the first case of onychomycosis described by Kligman in 1950 [67]. *Schizophyllum commune* is also well known as a plant parasitic fungus, aggressively colonizing trees in old alleys [68].

Previous studies have reported that environmental parameters such as temperature and relative humidity could affect the airborne fungal diversity [69]. According to our data analysis, there was a significant correlation between relative humidity and fungal communities, while the effect of temperature on the airborne fungal concentration and diversity was not significant. Our findings are consistent with a previous study conducted in Tianjin by Nageen et al. [26], which found that wind speed was the most critical factor influencing the fungal community in different outdoor environments, while temperature and relative humidity were less critical [26]. Conversely, other previous studies showed that temperature and humidity had a dominant effect on airborne fungal diversity [29,70]. For example, a study conducted in Hangzhou showed that air temperature could sustain the germination and growth of fungi, and therefore their presence in the air, in all seasons except winter [24]. Long-term data collection and large-scale studies are needed to better understand how different environmental factors affect the diversity and structure of airborne fungal communities.

Based on the analysis performed in our study, we did not find a significant difference in the distribution and relative abundance of major airborne fungal taxa at the two sites analyzed for each bridge, which were located at different heights. This finding is surprising as it contradicts what has been reported in some previous studies [71], and particularly in a study performed by Lu et al. [72] in one of the teaching and research buildings of Tianjin University [72]. The latter study revealed that the relative abundance of the major fungal taxa was affected by the height at different floors of the building [72]. Although our contradictory findings could be explained by the short distance between the ground level and the top of the analyzed pedestrian bridges, which is about 4.5 m, further studies are needed to clarify the influence of environmental factors that contributed to attenuate the effect of height on the fungal communities present at the C and U sites. However, looking in more detail at the results produced in this study, it is important to notice that, among the fungal species detected with a low percentage of abundance, some taxa known for their negative influences on human health were exclusively present in one of the two bridges’ sites. For instance, *Aspergillus flavus* and *A. niger* are well known pathogenic fungal species that were isolated only from the U sites of the bridges. *Aspergillus flavus* is an opportunistic animal and human pathogen that has been found in the air environments of several countries and described as a prevalent cause of invasive aspergillosis and toxic infections [73]. *Aspergillus flavus* produces aflatoxins that are commonly ingested through contaminated food from crops like groundnuts and maize, but can also be inhaled or come into contact with the skin or mucosa, thus causing aspergillosis diseases, especially in people with compromised immune systems [15,17,74,75,76,77,78]. This is particularly true for workers involved in the processing, storage, and transportation of crops, as they can be exposed to aflatoxin-contaminated dust [79]. Long-term exposure even to low levels of aflatoxins through either occupational inhalation or dietary consumption can lead to negative health effects, such as mucous membrane irritation, immune suppression, nausea, liver diseases, and cancer [79,80,81]. Inhalation of aflatoxins is more likely to result in respiratory-related cancers like alveolar cell carcinoma and lung cancer [15,82]. Furthermore, aflatoxin is responsible for the onset of aflatoxicosis, caused by ingestion of aflatoxin-contaminated food or by inhalation. This is a major issue in developing countries, particularly in Asia and Africa, where the consumption of mycotoxin contaminated maize has killed hundreds of people in recent years [76,78]. *Aspergillus niger* is considered a strong allergen and a pathogenic fungus that is typically connected to lung infections in people with weakened immune systems. Due to the production of small-sized conidia and conidiophores that can be easily inhaled, *A. niger* can cause systemic mycosis and invasive aspergillosis, including pulmonary disease, allergic bronchopulmonary disorder, and in some cases pneumonia [83,84,85]. Otomycosis and cutaneous infections have also been linked to *A. niger* [86,87]. Besides, *A. niger* produces ochratoxin A (OTA) and fumonisin B2 (FB_2_), which makes this fungal species a potentially harmful pathogenic contaminant of food, such as date-palm fruits [88]. Moreover, it is important to mention that, among the six *Aspergillus* species recorded in our study, only *A. sydowii* was shared by the two bridge sites, while the remaining five species, *A. aculeatus*, *A. flavus*, *A. flocculosus*, *A. niger*, and *A. versicolor* were only present in one site, which could make a significant difference in terms of human health risk. In fact, among the numerous pathogenic effects described for *Aspergillus* fungi, this fungal genus is known for its potential to induce infections in immunocompromised individuals and in patients with underlying pulmonary disease [89]. Fungal strains belonging to the genus *Trichoderma* were exclusively isolated from the C sites of the bridges. A number of *Trichoderma* species, including *T. harzianum* and *T. viride*, which were recorded in our study, have been described as human pathogenic fungi that could pose a serious health risk [90,91,92,93,94,95]. The genus *Aureobasidium*, exclusively detected in the U sites of the analyzed bridges, is another example of fungi that are noteworthy from the human health point of view. This fungal genus has been linked to subcutaneous phaeohyphomycosis observed in an immunocompetent carpenter in Morocco [72,96]. In the genus *Penicillium*, while the most abundant species, *P. oxalicum*, showed very similar distributions in the C and U sites, the two other recorded species, *P. brevicompactum* and *Penicillium* sp., were only detected at the sites under the studied bridges. This finding could represent important information for the assessment of health risk for people using bridges in urban environments, considering that the genus *Penicillium* is listed among the most common allergenic fungal taxa [27,97,98,99] and has been linked to asthma in previous studies [72,100,101].

Among those recorded fungi that contributed to differentiate the airborne fungal communities in the two investigated sites, at the top central area and along the sidewalk under the selected pedestrian bridges, we found some species that deserve to be mentioned, even if they are not supposed to have a direct harmful effect on pedestrians’ health. For instance, *Fusarium graminearum* is a common plant pathogenic ascomycete that was only isolated from the bridges’ C sites. *Fusarium* head blight (FHB), a plant disease caused by *F. graminearum*, is a worldwide issue causing significant financial impact on the cereal industry as it leads to lower grain yields and quality [102]. *Clonostachys rosea*, instead, was only detected in the U sites’ fungal communities. This ascomycetous fungal species, commonly recorded as a soil saprotroph, plant decomposer, and endophyte, was recently isolated for the first time as an entomopathogen of the Coleoptera species *Ophrida xanthospilota* (Chrysomelidae) in China [52].

## 5. Conclusions

This study provided a detailed assessment of the diversity of fungal communities at the top central area and along the sidewalk under the selected pedestrian bridges, which may represent an important source of information to understand the different exposure to microbial pollution for people walking in different points of the studied sites. While there was no significant difference in the distribution and abundance of the dominant airborne fungal taxa between the two investigated sites, it is noteworthy that, among the fungal species detected with a low percentage of abundance, some taxa known for their negative influences on human health were exclusively present in one of the two bridges’ sites. The fact that numerous species belonging to well-known pathogenic fungal genera, including *Alternaria*, *Aspergillus*, *Aureobasidium*, *Cladosporium*, *Penicillium*, and *Trichoderma*, were differently distributed at the two investigated bridge sites deserves further attention in order to clarify the human health risk associated with the inhalation of microbial particles in the analyzed sites. We clarified the role of environmental parameters (temperature and relative humidity) in shaping the analyzed fungal communities. In particular, the relative humidity showed a stronger influence compare to the temperature on the diversity and concentration of airborne fungi in the investigated sites. Such influence was either positive or negative on different fungal genera. This result suggests that environmental factors could be consider to predict the composition of fungal communities in different periods of the day and in different seasons, in order to assess the related health risk for people using pedestrian bridges in urban environments. Further studies are still needed to disentangle the effects of different environmental factors on the diversity and structure of airborne fungi. The most abundant fungi found in our work belong to the genera *Alternaria* and *Cladosporium*, which are known for their potentially hazardous impacts on human health, and deserve particular attention and monitoring in order to prevent airborne fungi-related diseases. The results of this study may provide valuable information for air quality monitoring, airborne disease prevention, and microbial pollution control, and could be particularly helpful for assessing human health risks.

## Figures and Tables

**Table 1:** Fungal concentration recorded at the different analyzed sites (mean = average fungal concentration, SD = standard deviation).

Sampling Sites		Mean (CFU/m^3^)	SD	Min (CFU/m^3^)	Max (CFU/m^3^)
BJD	C	70.00	26.46	40	90
	U	103.33	70.95	40	180
NG	C	90.00	26.46	70	120
	U	63.33	35.12	30	100
WJY	C	63.33	55.08	10	120
	U	103.33	37.86	60	130
WJR	C	60.00	26.46	40	90
	U	60.00	55.68	10	120
NMWA	C	90.00	72.11	10	150
	U	56.67	5.77	50	60

**Table 2:** Shannon index of fungal community diversity in different sampling locations for each sampling week.

Sampling Locations	BJD		NG		WJY		WJR		NMWA	
	C	U	C	U	C	U	C	U	C	U
First week	0.79	1.04	1.15	1.1	0	1.56	1.61	0	0	1.33
Second week	1.38	1.52	1.91	1.56	2.14	1.93	1.04	2.37	2.21	1.56
Third week	1.43	1.35	1.79	1.97	1.33	1.31	1.43	1.61	1.12	1.79

## Data Availability

Data presented in this study can be found as part of the manuscript, and in the Supplementary Materials. The fungal DNA sequences amplified during this study are available at GenBank under accessions ON705346–ON705573.

## Supplementary Materials

The following supporting information can be downloaded at: https://www.mdpi.com/article/10.3390/microorganisms11082097/s1, Table S1: Details of the selected sampling sites; Table S2: Airborne fungal diversity molecularly detected in the five studied pedestrian bridges, from DNA extracted from isolated strains; Table S3: Distribution of the most abundant fungal genera. Genera with very low relative abundances (<1%) were merged as “others”; Table S4: Airborne fungal concentration at each sampling location; Table S5: Fungal genera distribution at U and C sites; Table S6: Environmental factors recorded in each sampling site at the time of sampling (C = center of the bridge; U = under the bridge). Figure S1: Sampling sites. NG (a), NMWA (b), and WJR pedestrian bridges; top central area of BJD pedestrian bridge (d). Figure S2: Collection of air samples (a,b) and examples of airborne fungal strains isolated from the analyzed pedestrian bridges (c,d). Figure S3: Fungal concentration at each experimental location (C = the top central area of the bridges; U = under the bridges along the side walk). Figure S4: Concentration of airborne fungi at the center and under the 5 bridges during the 3 weeks sampling period (CFU/m^3^). Figure S5: Concentration of the five most abundant fungal genera collected from the two analyzed sites (C = the top central area of the bridges; U = under the bridges along the side walk) at each pedestrian bridge. Figure S6: Total number of fungal genera shared by both sites (C = the top central area of the bridges; U = under the bridges along the side walk) selected for each bridge. Figure S7: Variation of ten most abundant fungal genera in the two sites at the center and under the investigated bridges, based on Wilcoxon rank-sum test. Error bars represent SEM.
