# Supplementary material for: Analysis of Airborne Fungal Communities on Pedestrian Bridges in Urban Environments
Source: Microorganisms. 2023 Aug 16;11(8):2097. doi: 10.3390/microorganisms11082097 (PMC10458245; doi:10.3390/microorganisms11082097)
Supplement: Supplementary file 1 [file microorganisms-11-02097-s001.zip › microorganisms-2458347-supplementary.pdf]

Analysis of Airborne Fungal Communities on Pedestrian Bridges in Urban Environments

Amran A. Q. A. Al-Shaarani, Ziwei M. Quach, Xiao Wang †, Mohammed H. M. Muafa, Md M. H. Nafis and Lorenzo Pecoraro *

School of Pharmaceutical Science and Technology, Tianjin University, 92 Weijin Road, Tianjin 300072, China; amran303@163.com (A.A.Q.A.A.-S.); ziweimelaniequach@tju.edu.cn (Z.M.Q.); wang_xiao1996@163.com (X.W.); muafa99@tju.edu.cn (M.H.M.M.); mh.nafis29oct@gmail.com (M.M.H.N.)

* Correspondence: lorenzo.pecoraro@tju.edu.cn; Tel.: +86-185-2082-4550

† Current address: Department of Ocean Science, The Hong Kong University of Science and Technology, Hong Kong SAR, China.

Supplementary information

Supplementary Table S1. Details of the selected sampling sites.

Weijin road pedestrian bridge (WJR)	
**Sampling site**	**GPS**
Top [center of the bridge (C)]	39°6’10”N 117°10’22”E
Down [under the bridge (U)]	39°6’10”N 117°10’22”E
**Tianjin university old campus Northgate pedestrian bridge (NG)**	
**Sampling site**	**GPS**
Top [center of the bridge (C)]	39°6’35”N 117°10’7”E
Down [under the bridge (U)]	39°6’30”N 117°10’11”E
**Binjiangdao pedestrian bridge (BJD)**	
**Sampling site**	**GPS**
Top [center of the bridge (C)]	39°7’30”N 117°11’14”E
Down [under the bridge (U)]	39°7’30”N 117°11’13”E
**Nanmenwai avenue pedestrian bridge (NMWA)**	
**Sampling site**	**GPS**
Top [center of the bridge (C)]	39°7’35”N 117°10’26”E
Down [under the bridge (U)]	39°7’35”N 117°10’25”E
**Wujiayao avenue pedestrian bridge (WJY)**	
**Sampling site**	**GPS**
Top [center of the bridge (C)]	39°5’58”N 117°10’56”E
Down [under the bridge (U)]	39°5’58”N 117°10’57”E

Supplementary Table S2: Airborne fungal diversity molecularly detected in the five studied pedestrian bridges, from DNA extracted from isolated strains.

Sample No.	Sampling week	Sampling site	GenBank	Best BLAST match(es)	Accession code	Overlap length	Query curve	Match %
1	1st week	NMWA (UN)	ON705346	*Penicillium* sp.	MN634686.1	539	94%	99.63%
				Penicillium thomii	MG818940.1	551	95%	99.63%
2	1st week	NMWA (UN)	ON705347	*Alternaria* sp.	MT735240.1	597	96%	100.00%
				Alternaria alternata	MN594810.1	598	93%	99.81%
3	1st week	NMWA (UN)	ON705348	*Arthrinium* sp.	KJ361492.1	589	93%	100.00%
				Arthrinium arundinis	LT719147.1	1070	93%	99.62%
4	1st week	NMWA (UN)	ON705349	*Alternaria alternata*	MW009041.1	542	94%	100.00%
5	1st week	NMWA (UN)	ON705350	*Aspergillus sydowii*	MT582755.1	627	100%	99.07%
6	1st week	NMWA (UN)	ON705351	*Sporobolomyces beijingensis*	MK592821.1	560	94%	99.82%
7	1st week	NG (UN)	ON705352	*Sporobolomyces beijingensis*	ON705352.1	676	100%	100.00%
8	1st week	NG (UN)	ON705353	*Sporidiobolus pararoseus*	MN956990.1	586	100%	100.00%
9	1st week	WJR (C)	ON705354	*Keissleriella* sp.	ON705354.1	560	100%	100.00%
				Keissleriella caraganae	NR_164447.1	522	89%	98.81%
10	1st week	WJR (C)	ON705355	*Lophiostoma corticola*	ON705355.1	568	100%	100.00%
11	1st week	WJR (C)	ON705356	*Coprinellus radians*	MK087751.1	729	91%	99.84%
12	1st week	WJR (C)	ON705360	*Bipolaris setariae*	HE792936.1	575	93%	99.82%
13	1st week	WJR (C)	ON705361	Dothideomycetes sp.	JQ759885.1	1080	100%	100.00%
				Periconia sp.	MW391746.1	540	91%	99.60%
14	1st week	NG (C)	ON705362	*Aspergillus versicolor*	KX349474.1	545	93%	99.81%
15	1st week	NG (C)	ON705360	*Didymella glomerata*	MH791089.1	521	93%	99.03%
16	1st week	NG (C)	ON705361	Ascomycota sp.	JN120374.1	1094	95%	93.39%
				Paraphoma fimeti	MW221097.1	574	87%	92.46%
17	1st week	NG (C)	ON705362	*Aspergillus* sp.	KF964033.1	541	95%	99.62%
18	1st week	NG (C)	ON705363	*Phoma fungicola*	KF293780.1	515	95%	99.81%
19	1st week	NG (C)	ON705364	*Aspergillus sydowii*	MH712267.1	565	95%	99.44%
20	1st week	NG (C)	ON705365	*Aspergillus sydowii*	JX518253.1	540	96%	99.08%
21	1st week	WJY (UN)	ON705366	*Didymella* sp.	MG198901.1	534	100%	100.00%
				Didymella glomerata	ON705360.1	547	96%	99.22%
22	1st week	WJY (UN)	ON705367	*Rhizopus oryzae*	MK246921.1	602	99%	99.66%
23	1st week	WJY (UN)	ON705368	*Cladosporium halotolerans*	MT588810.1	575	93%	99.60%
24	1st week	WJY (UN)	ON705369	*Scopulariopsis brevicaulis*	MF044048.1	580	96%	97.06%
25	1st week	WJY (UN)	ON705370	*Cladosporium cladosporioides*	KU645993.1	561	95%	94.74%
26	1st week	WJY (UN)	ON705371	*Penicillium brevicompactum*	KX067822.1	1046	95%	99.64%
27	1st week	BJD (UN)	ON705372	*Cladosporium cladosporioides*	MW113324.1	523	96%	99.23%

28	1st week	BJD (UN)	ON705373	*Alternaria compacta*	MW009019.1	553	98%	99.81%
29	1st week	BJD (UN)	ON705374	*Aspergillus flocculosus*	MH101466.1	796	96%	99.13%
30	1st week	BJD (UN)	ON705375	*Cladosporium tenuissimum*	MH270558.1	516	94%	100.00%
31	1st week	BJD (C)	ON705376	*Trichoderma* sp.	MK870316.1	581	98%	99.65%
32	1st week	BJD (C)	ON705377	*Trichoderma* sp.	MK871149.1	580	98%	99.48%
33	1st week	BJD (C)	ON705378	*Alternaria tenuissima*	MZ160946.1	588	98%	99.45%
34	1st week	BJD (C)	ON705379	*Alternaria tamaricis*	MW449616.1	539	97%	99.81%
35	1st week	BJD (C)	ON705380	*Alternaria alternata*	MW009000.1	553	98%	99.45%
36	1st week	BJD (C)	ON705381	*Alternaria tenuissima*	MZ160951.1	539	96%	99.81%
37	1st week	BJD (C)	ON705382	*Chaetomium cruentum*	JN209871.1	1140	98%	98.57%
38	1st week	BJD (C)	ON705383	*Trichoderma viride*	HM037928.1	581	97%	99.29%
39	1st week	WJY (C)	ON705384	*Moesziomyces bullatus*	MK024203.1	755	99%	99.45%
40	1st week	NMWA (C)	ON705385	*Choanephora infundibulifera*	MH856792.1	635	94%	99.33%
41	1st week	WJR (UN)	ON705386	*Alternaria alternata*	MT487774.1	551	94%	99.81%
42	2nd week	BJD (UN)	ON705387	Pleosporales sp.	MT974576.1	569	92%	99.10%
43	2nd week	BJD (UN)	ON705388	*Filobasidium magnum*	MK592828.1	596	92%	99.66%
44	2nd week	BJD(UN)	ON705389	*Sporidiobolus pararoseus*	MN956990.1	1156	88%	99.64%
45	2nd week	BJD (UN)	ON705390	*Schizophyllum* sp.	MK732120.1	633	83%	98.32%
				Schizophyllum commune	MK680083.1	680	83%	98.31%
46	2nd week	BJD (UN)	ON705391	*Sporidiobolus ruineniae*	HQ670680.1	609	90%	97.88%
47	2nd week	BJD (UN)	ON705392	*Schizophyllum commune*	MF061788.1	606	100%	99.18%
48	2nd week	BJD (UN)	ON705393	*Alternaria tenuissima*	MW723827.1	551	92%	93.20%
49	2nd week	WJR (C)	ON705394	*Sporobolomyces beijingensis*	NR_137663.1	638	97%	99.64%
50	2nd week	WJR (C)	ON705395	*Schizophyllum commune*	MF061788.1	606	99%	99.83%
51	2nd week	WJR (C)	ON705396	*Keissleriella* sp.	MW764302.1	543	96%	99.44%
52	2nd week	WJR (C)	ON705397	*Schizophyllum commune*	KU042974.1	614	93%	98.63%
53	2nd week	BJD (C)	ON705398	*Hannaella oryzae*	KY103504.1	646	98%	99.13%
54	2nd week	BJD (C)	ON705399	*Sporidiobolus pararoseus*	MN956990.1	1156	95%	99.82%
55	2nd week	BJD (C)	ON705400	*Coprinellus radians*	MK732139.1	672	92%	98.83%
56	2nd week	BJD (C)	ON705401	*Sporobolomyces carnicolor*	MT529926.1	608	93%	94.30%
57	2nd week	BJD (C)	ON705402	*Hannaella oryzae*	KY103504.1	646	68%	92.58%
58	2nd week	NG (C)	ON705403	*Alternaria tenuissima*	MW723827.1	551	89%	97.09%
59	2nd week	NG (C)	ON705404	*Sporobolomyces carnicolor*	MK592826.1	571	97%	98.39%
60	2nd week	NG (C)	ON705405	*Schizophyllum commune*	HQ331059.1	597	75%	98.93%
61	2nd week	NG (C)	ON705406	*Irpex lacteus*	KX958063.1	1691	97%	99.06%
62	2nd week	NG (C)	ON705407	*Coprinellus radians*	KP900252.1	658	99%	96.49%
63	2nd week	NG (C)	ON705408	*Schizophyllum* sp.	MZ501801.1	600	95%	99.66%
				Schizophyllum commune	MN218205.1	639	95%	99.49%

64	2nd week	WJY (C)	ON705409	*Hypocreales* sp.	MN856379.1	545	93%	95.59%
65	2nd week	WJY (C)	ON705410	*Sporobolomyces beijingensis*	MK592821.1	560	86%	93.36%
66	2nd week	WJY (C)	ON705411	*Sporidiobolus pararoseus*	MN956990.1	1156	95%	99.64%
67	2nd week	WJY (C)	ON705412	*Sporidiobolus pararoseus*	ON705412.1	622	100%	100.0%
68	2nd week	WJY (C)	ON705413	*Typhula micans*	ON705413.1	129	100%	100.0%
69	2nd week	WJY (C)	ON705414	Pleosporales sp.	MK595645.1	723	96%	99.60%
				Parastagonospora nodorum	KF512822.1	1034	96%	99.60%
70	2nd week	WJY (C)	ON705415	*Botryosporium* sp.	MH428684.1	479	78%	89.94%
				Botryosporium longibrachiatum	KF372591.1	604	88%	91.96%
71	2nd week	WJY (C)	ON705416	*Hannaella oryzae*	KY103504.1	646	96%	99.12%
72	2nd week	WJY (C)	ON705417	*Sporobolomyces carnicolor*	MT529870.1	557	98%	99.64%
73	2nd week	WJY (C)	ON705418	*Hannaella oryzae*	KY103504.1	646	96%	97.60%
74	2nd week	WJY (C)	ON705419	Basidiomycota sp.	MF680430.1	628	95%	99.84%
				Funalia trogii	EF546236.1	646	95%	100.00%
75	2nd week	WJY (C)	ON705420	*Cladosporium* sp.	MG554329.1	587	90%	99.04%
				Cladosporium parahalotolerans	MF473164.1	837	90%	99.04%
76	2nd week	WJR (UN)	ON705421	*Sporobolomyces carnicolor*	KY962981.1	603	96%	99.30%
77	2nd week	WJR (UN)	ON705422	*Penicillium oxalicum*	MH766384.1	564	98%	100.00%
78	2nd week	WJR (UN)	ON705423	*Coprinellus radians*	MK087751.1	729	96%	99.69%
79	2nd week	WJR (UN)	ON705424	Ascomycota sp.	JN120374.1	1094	89%	93.95%
80	2nd week	WJR (UN)	ON705425	*Rhodosporidiobolus odoratus*	MT340844.1	581	96%	99.65%
81	2nd week	WJR (UN)	ON705426	*Coprinellus radians*	MK087751.1	729	96%	99.69%
82	2nd week	WJR (UN)	ON705427	*Sporobolomyces carnicolor*	MK592826.1	571	96%	99.30%
83	2nd week	WJR (UN)	ON705428	*Coprinellus radians*	MK087751.1	729	95%	99.39%
84	2nd week	WJR (UN)	ON705429	*Hirsutella* sp.	MZ380071.1	587	94%	99.81%
85	2nd week	WJR (UN)	ON705430	*Psathyrella bivelata*	MF325962.1	728	94%	99.85%
86	2nd week	WJR (UN)	ON705431	*Colletotrichum gloeosporioides*	JQ356541.1	551	95%	99.62%
87	2nd week	WJR (UN)	ON705432	*Aureobasidium pullulans*	MN922096.1	543	71%	98.77%
88	2nd week	WJR (UN)	ON705433	Hypocreales sp.	MN856379.1	545	97%	99.07%
				Clonostachys rosea	MW113490.1	588	95%	99.81%
89	2nd week	WJR (UN)	ON705434	*Keissleriella* sp.	MW764302.1	543	96%	99.07%
90	2nd week	WJY (UN)	ON705435	*Coprinellus xanthothrix*	JN198387.1	700	97%	99.24%
91	2nd week	WJY (UN)	ON705436	*Sporobolomyces carnicolor*	KY962981.1	603	94%	98.76%
92	2nd week	WJY (UN)	ON705437	*Aureobasidium pullulans*	DQ680843.1	555	97%	98.20%
93	2nd week	WJY (UN)	ON705438	*Sporidiobolus* sp.	KJ908838.1	577	97%	99.29%
				Sporobolomyces carnicolor	MT529870.1	557	95%	99.63%
94	2nd week	WJY(UN)	ON705439	*Aureobasidium pullulans*	DQ680843.1	555	94%	98.20%
95	2nd week	WJY(UN)	ON705440	*Schizophyllum commune*	MH392740.1	648	94%	99.50%

96	2nd week	WJY (UN)	ON705441	*Sporidiobolus* sp.	KJ908838.1	577	98%	99.47%
				Sporobolomyces carnicolor	MT529870.1	557	96%	99.64%
97	2nd week	WJY (UN)	ON705442	*Phaeosphaeria* sp.	MH884074.1	521	93%	99.42%
				Phaeosphaeria fuckelii	MZ396933.1	563	95%	98.86%
98	2nd week	WJY (UN)	ON705443	*Bullera* sp.	HQ631046.1	514	95%	99.10%
				Hannaella sinensis	NR_111075.1	474	95%	99.10%
99	2nd week	WJY (UN)	ON705444	*Papiliotrema fuscus*	KY962979.1	572	95%	98.87%
100	2nd week	NMWA (UN)	ON705445	*Coprinellus radians*	KP900252.1	658	98%	99.54%
101	2nd week	NMWA (UN)	ON705446	*Schizophyllum commune*	MH483677.1	614	99%	99.33%
102	2nd week	WJR (UN)	ON705447	*Irpex lacteus*	MT085750.1	643	99%	99.68%
103	2nd week	NMWA (UN)	ON705448	*Sporidiobolus pararoseus*	MN340037.1	571	95%	99.28%
104	2nd week	NMWA (UN)	ON705449	*Schizophyllum commune*	MN523303.1	592	97%	97.30%
105	2nd week	NMWA (UN)	ON705450	*Sporobolomyces carnicolor*	MK592826.1	571	94%	99.64%
106	2nd week	NMWA (UN)	ON705451	*Cochliobolus sativus*	JQ754031.1	580	96%	99.82%
107	2nd week	NMWA (C)	ON705452	*Cyphellaceae* sp.	MG269994.1	702	95%	99.85%
108	2nd week	NMWA (C)	ON705453	*Sporidiobolus pararoseus*	MN340037.1	571	95%	99.11%
109	2nd week	NMWA (C)	ON705454	*Coprinopsis atramentaria*	MG132086.1	681	96%	98.96%
110	2nd week	NMWA (C)	ON705455	*Fusarium graminearum*	MF800908.1	517	79%	99.29%
111	2nd week	NMWA (C)	ON705456	*Irpex lacteus*	MT085750.1	643	96%	99.38%
112	2nd week	NMWA (C)	ON705457	*Irpex lacteus*	ON705457.1	1241	100%	100.0%
113	2nd week	NMWA (C)	ON705458	*Sporobolomyces carnicolor*	KY962981.1	603	98%	99.46%
114	2nd week	NMWA (C)	ON705459	*Papiliotrema fuscus*	KY962979.1	572	95%	98.68%
115	2nd week	NMWA (C)	ON705460	*Colletotrichum gloeosporioides*	JQ356541.1	551	98%	98.91%
116	2nd week	NMWA (C)	ON705461	*Sporobolomyces carnicolor*	MK592826.1	571	97%	99.47%
117	2nd week	NMWA (C)	ON705462	*Schizophyllum commune*	MF326432.1	647	76%	98.95%
118	2nd week	NMWA (C)	ON705463	*Schizophyllum commune*	MF061788.1	606	99%	99.66%
119	2nd week	NMWA (C)	ON705464	*Fusarium graminearum*	MK762537.1	503	68%	98.44%
120	2nd week	NMWA (C)	ON705465	*Sporidiobolus pararoseus*	MN340037.1	571	95%	99.28%
121	2nd week	NMWA (C)	ON705466	*Fusarium graminearum*	MZ648312.1	517	96%	99.61%
122	2nd week	NMWA (C)	ON705467	*Schizophyllum commune*	MF061788.1	606	98%	99.01%
123	2nd week	NG (UN)	ON705468	*Pyricularia urashimae*	MT586118.1	510	94%	99.80%
124	2nd week	NG (UN)	ON705469	*Clonostachys rosea*	MW113490.1	588	95%	99.62%
125	2nd week	NG (UN)	ON705470	*Cladosporium* sp.	MZ452398.1	546	97%	99.61%
				Cladosporium cladosporioides	MT878604.1	538	97%	99.42%
126	2nd week	NG (UN)	ON705471	Cyphellaceae sp.	MG269994.1	702	93%	99.54%
127	2nd week	NG (UN)	ON705472	*Sporobolomyces carnicolor*	KY962981.1	603	94%	99.64%
128	2nd week	NG (UN)	ON705473	*Schizophyllum commune*	MF061788.1	606	98%	99.01%
129	2nd week	NG (UN)	ON705474	Pleosporales sp.	HQ631015.1	588	79%	98.58%

130	2nd week	NG (UN)	ON705475	*Neosetophoma* sp.	KU751879.1	569	96%	99.82%
131	3rd week	NG (C)	ON705476	*Cladosporium tenuissimum*	JN624886.1	518	98%	99.81%
132	3rd week	NG (C)	ON705477	*Moesziomyces aphidis*	MN038047.1	812	98%	99.60%
133	3rd week	NG (C)	ON705478	*Trichothecium roseum*	MH470248.1	603	97%	99.14%
134	3rd week	NG (C)	ON705479	*Magnaporthe* sp.	MK595550.1	731	97%	98.84%
				Pyricularia grisea	OK093407.1	577	95%	99.41%
135	3rd week	NG (C)	ON705480	*Cladosporium halotolerans*	MT588810.1	575	98%	95.80%
136	3rd week	NG (C)	ON705480	*Cercospora brassicicola*	MN209930.1	547	97%	99.41%
137	3rd week	NG (C)	ON705482	*Cladosporium cladosporioides*	MW113635.1	669	98%	99.07%
138	3rd week	NG (C)	ON705483	*Alternaria brassicae*	MW008888.1	545	98%	99.63%
139	3rd week	NG (C)	ON705484	*Cladosporium tenuissimum*	KP689183.1	522	98%	99.61%
140	3rd week	NG (C)	ON705485	*Alternaria* sp.	MT487763.1	545	96%	99.81%
				Alternaria tenuissima	KR709056.1	545	96%	100.00%
141	3rd week	NG (C)	ON705486	*Penicillium oxalicum*	MT597864.1	601	77%	99.31%
142	3rd week	NG (C)	ON705487	*Cercospora canescens*	MN400244.1	547	99%	99.02%
143	3rd week	NG (UN)	ON705488	*Curvularia plantarum*	MW581905.1	606	98%	99.47%
144	3rd week	NG (UN)	ON705489	*Rhodotorula mucilaginosa*	MN427959.1	1140	98%	98.82%
145	3rd week	NG (UN)	ON705490	*Rhodotorula* sp.	MW008901.1	543	97%	99.81%
				Alternaria compacta	MW008904.1	542	97%	99.63%
146	3rd week	NG (UN)	ON705491	*Penicillium oxalicum*	KY400080.1	574	98%	99.29%
147	3rd week	NG (UN)	ON705492	*Cladosporium oxysporum*	MT079170.1	526	99%	99.42%
148	3rd week	NG (UN)	ON705493	*Magnaporthe oryzae*	FN555113.1	560	97%	98.65%
149	3rd week	NG (UN)	ON705494	*Cladosporium tenuissimum*	KP689183.1	522	96%	99.61%
150	3rd week	NG (UN)	ON705495	*Pseudopithomyces palmicola*	MN788110.1	848	97%	98.98%
151	3rd week	NG (UN)	ON705496	*Cladosporium cladosporioides*	MW453186.1	525	97%	99.42%
152	3rd week	NG (UN)	ON705497	*Aspergillus niger*	MW958037.1	586	96%	99.82%
153	3rd week	WJR (C)	ON705498	*Alternaria alternata*	MF099863.1	982	99%	99.27%
154	3rd week	WJR (C)	ON705499	*Cladosporium cladosporioides*	MW113635.1	669	98%	98.52%
155	3rd week	WJR (C)	ON705500	*Alternaria alternata*	MW008971.1	542	98%	99.63%
156	3rd week	WJR (C)	ON705501	*Alternaria compacta*	MW008978.1	537	97%	100.00%
157	3rd week	WJR (C)	ON705502	*Nigrospora* sp.	MN341508.1	555	84%	99.34%
				Nigrospora oryzae	MF356577.1	532	96%	99.34%
158	3rd week	WJR (C)	ON705503	*Cladosporium oxysporum*	MT079170.1	526	96%	99.41%
159	3rd week	WJR (C)	ON705504	*Alternaria alternata*	MT093259.1	546	97%	99.44%
160	3rd week	WJR (C)	ON705505	*Penicillium oxalicum*	MN856268.1	594	87%	100.00%
161	3rd week	WJR (C)	ON705506	*Moesziomyces aphidis*	MH777069.1	793	33%	93.44%
162	3rd week	WJR (UN)	ON705507	*Cladosporium tenuissimum*	MT072081.1	525	98%	99.42%

163	3rd week	WJR (UN)	ON705508	Ascomycota sp.	MH430722.1	551	98%	99.63%
164	3rd week	WJR (UN)	ON705509	*Aspergillus aculeatus*	MK450627.1	851	82%%	99.12%
165	3rd week	WJR (UN)	ON705510	*Alternaria* sp.	MK649975.1	544	98%	99.81%
				Alternaria brassicae	MW008950.1	553	98%	100.00%
166	3rd week	WJR (UN)	ON705511	*Epicoccum nigrum*	MH645206.1	586	97%	99.23%
167	3rd week	WJY (C)	ON705512	*Alternaria alternata*	KF293886.1	545	100%	99.26%
168	3rd week	WJY (C)	ON705513	*Penicillium oxalicum*	MT597864.1	601	90%	100.00%
169	3rd week	WJY (C)	ON705514	*Alternaria alternata*	MW008892.1	542	97%	99.63%
170	3rd week	WJY (C)	ON705515	*Septoriella oudemansii*	KR873250.1	1142	93%	100.00%
171	3rd week	WJY (C)	ON705516	*Trichoderma harzianum*	KJ767088.1	622	99%	92.39%
172	3rd week	WJY (C)	ON705517	*Trichoderma harzianum*	MW454982.1	584	96%	99.66%
173	3rd week	WJY (UN)	ON705518	*Amphobotrys ricini*	MT223777.1	489	83%	99.74%
174	3rd week	WJY (UN)	ON705519	*Pyricularia urashimae*	MT586118.1	510	95%	99.21%
175	3rd week	WJY (UN)	ON705520	*Pleosporales* sp.	KX379244.1	525	95%	99.81%
				Cladosporium cladosporioides	KX258800.1	526	96%	99.43%
176	3rd week	WJY (UN)	ON705521	*Cladosporium cladosporioides*	KF876823.1	522	97%	99.42%
177	3rd week	WJY (UN)	ON705522	*Alternaria angustiovoidea*	MW009022.1	551	97%	100.00%
178	3rd week	WJY (UN)	ON705523	*Alternaria compacta*	MW008933.1	540	97%	99.44%
179	3rd week	WJY (UN)	ON705524	*Cladosporium cladosporioides*	KF876823.1	522	97%	99.42%
180	3rd week	WJY (UN)	ON705525	*Cladosporium oryzae*	KY400092.1	532	97%	99.43%
181	3rd week	WJY (UN)	ON705526	*Alternaria solani*	MW009039.1	551	98%	99.64%
182	3rd week	WJY (UN)	ON705527	*Alternaria* sp.	MK649973.1	543	98%	99.63%
				Alternaria tamaricis	MW009013.1	542	97%	99.81%
183	3rd week	WJY (UN)	ON705528	*Alternaria solani*	MW009039.1	551	97%	99.82%
184	3rd week	WJY (UN)	ON705529	*Alternaria alternata*	MW008996.1	548	98%	99.45%
185	3rd week	NMWA (C)	ON705530	*Alternaria* sp.	KX064983.1	544	97%	99.63%
				Alternaria tenuissima	KM513592.1	547	97%	99.45%
186	3rd week	NMWA (C)	ON705531	*Alternaria brassicae*	MW009012.1	552	97%	99.81%
187	3rd week	NMWA (C)	ON705532	*Cladosporium cladosporioides*	KC880082.1	525	97%	99.81%
188	3rd week	NMWA (C)	ON705533	*Alternaria alternata*	MN173818.1	542	97%	99.63%
189	3rd week	NMWA (C)	ON705534	*Alternaria alternata*	MK281551.1	546	97%	99.45%
190	3rd week	NMWA (C)	ON705535	*Alternaria solani*	MW009039.1	551	97%	100.00%
191	3rd week	NMWA (C)	ON705536	*Deniquelata* sp.	MH316153.1	686	93%	98.25%
				Deniquelata quercina	MT820404.1	675	90%	98.79%
192	3rd week	NMWA (C)	ON705537	*Alternaria* sp.	MK649974.1	545	97%	100.00%
193	3rd week	NMWA (C)	ON705538	*Alternaria angustiovoidea*	MW009022.1	551	96%	99.81%
194	3rd week	NMWA (C)	ON705539	*Cladosporium cucumerinum*	KR912311.1	516	94%	100.00%
195	3rd week	NMWA (C)	ON705540	*Moesziomyces bullatus*	MK024203.1	755	96%	90.96%

196	3rd week	NMWA (C)	ON705541	*Cladosporium tenuissimum*	JN624886.1	518	96%	99.61%
197	3rd week	NMWA (UN)	ON705542	*Fusarium incarnatum*	MT565585.1	505	70%	98.63%
198	3rd week	NMWA (UN)	ON705543	*Pyricularia urashimae*	MT586118.1	510	79%	98.58%
199	3rd week	NMWA (UN)	ON705544	*Pyricularia urashimae*	ON705544.1	567	100.00%	99.46%
200	3rd week	NMWA (UN)	ON705545	*Aspergillus flavus*	MF685316.1	555	96%	98.19%
201	3rd week	NMWA (UN)	ON705546	*Ascomycota* sp.	MH430722.1	551	90%	99.19%
				Aspergillus aculeatus	MH389050.1	552	90%	99.19%
202	3rd week	BJD (C)	ON705547	*Alternaria* sp.	MK649963.1	548	96%	99.81%
				Alternaria compacta	MW009019.1	553	96%	99.81%
203	3rd week	BJD (C)	ON705548	*Torula* sp.	KC427082.1	567	95%	99.62%
204	3rd week	BJD (C)	ON705549	*Alternaria solani*	MW009039.1	551	99%	99.63%
205	3rd week	BJD (C)	ON705550	*Alternaria* sp.	ON705550.1	558	100%	100.00%
				Alternaria compacta	ON790467.1	678	97%	99.82%
206	3rd week	BJD (C)	ON705551	*Alternaria* sp.	ON705551.1	555	100%	100.00%
				Alternaria compacta	OP364483.1	554	96%	100.00%
207	3rd week	BJD (C)	ON705552	*Cladosporium* sp.	ON705552.1	535	100%	100.00%
				Cladosporium tenuissimum	OM237117.1	533	96%	99.81%
208	3rd week	BJD (C)	ON705553	*Phoma multirostrata*	KR709058.1	530	96%	99.62%
209	3rd week	BJD (C)	ON705554	*Penicillium oxalicum*	MH367526.1	564	100%	99.29%
210	3rd week	BJD (C)	ON705555	*Alternaria angustiovoidea*	MW009022.1	551	97%	99.45%
211	3rd week	BJD (C)	ON705556	*Cladosporium cladosporioides*	MT367262.1	556	87%	99.33%
212	3rd week	BJD (C)	ON705557	*Alternaria solani*	MN871616.1	541	88%	99.38%
213	3rd week	BJD (UN)	ON705558	*Alternaria alternata*	KX783402.1	545	99%	99.81%
214	3rd week	BJD (UN)	ON705559	*Cladosporium oxysporum*	MK226241.1	522	82%	98.84%
215	3rd week	BJD (UN)	ON705560	*Cladosporium sphaerospermum*	MT582795.1	1369	72%	98.96%
216	3rd week	BJD (UN)	ON705561	*Pseudozyma* sp.	MN515019.1	780	97%	100.00%
				Moesziomyces antarcticus	MK027038.1	747	97%	100.00%
217	3rd week	BJD (UN)	ON705562	*Penicillium oxalicum*	MH766384.1	564	98%	100.00%
218	3rd week	BJD (UN)	ON705563	*Alternaria alternata*	MW009036.1	552	98%	99.81%
219	3rd week	BJD (UN)	ON705564	*Alternaria alternata*	MW008882.1	543	98%	99.07%
220	3rd week	BJD (UN)	ON705565	*Alternaria tenuissima*	MK534905.1	528	72%	99.24%
221	3rd week	BJD (UN)	ON705566	*Cercospora chrysanthemi*	KR709060.1	515	99%	99.80%
222	3rd week	BJD (UN)	ON705567	*Alternaria* sp.	MK649972.1	547	99%	99.44%
				Alternaria tenuissima	KX783378.1	543	100%	99.44%
223	3rd week	BJD (UN)	ON705568	*Cercospora capsici*	HQ700354.1	513	98%	99.60%
224	3rd week	BJD (UN)	ON705569	*Alternaria alternata*	MT635274.1	566	96%	100.00%
225	3rd week	BJD (UN)	ON705570	*Bipolaris setariae*	MN215638.1	722	97%	99.45%

226	3rd week	BJD (UN)	ON705571	*Cladosporium delicatulum*	MN644691.1	1071	90%	99.58%
227	3rd week	BJD (UN)	ON705572	*Alternaria porri*	MW009048.1	552	98%	100.00%
228	3rd week	BJD (UN)	ON705573	*Alternaria tenuissima*	MH824253.1	570	94%	99.42

Supplementary Table S3: Distribution of the most abundant fungal genera. Genera with very low relative abundances (<1%) were merged as "others".

	BJD		NG		WJY		WJR		NMWA		Total	%
	C	U	C	U	C	U	C	U	C	U		
Alternaria	8	12	3	1	2	6	4	2	6	3	47	20.61
Ascomycota	0	0	0	0	0	0	0	2	0	1	3	1.32
Aspergillus	0	1	4	1	0	0	0	1	0	2	9	3.95
Aureobasidium	0	0	0	0	0	2	0	1	0	1	4	1.75
Cercospora	0	2	2	0	0	0	0	0	0	0	4	1.75
Cladosporium	1	6	5	3	1	5	2	1	3	0	27	11.84
Coprinellus	1	0	1	0	0	4	1	0	0	1	8	3.51
Fusarium	0	0	0	0	0	0	0	0	3	1	4	1.75
Hannaella	1	0	1	0	2	0	0	0	0	0	4	1.75
Irpex	0	0	1	0	0	0	0	1	1	0	3	1.32
Keissleriella	0	0	0	0	0	0	2	1	0	0	3	1.32
Moesziomyces	0	0	1	0	1	0	1	0	1	0	4	1.75
Penicillium	1	1	1	1	1	1	1	1	0	1	9	3.95
Pyricularia	0	0	0	1	0	1	0	0	0	1	3	1.32
Schizophyllum	0	2	2	2	1	1	2	0	2	2	14	6.14
Sporidiobolus	1	3	0	1	1	2	0	0	2	1	11	4.82
Sporobolomyces	1	0	1	2	2	1	1	2	2	1	13	5.70
Trichoderma	3	0	0	0	2	0	0	0	0	0	5	2.19
Others (<1%)	3	3	4	6	6	6	4	6	6	2	46	20.18

Supplementary Table S4: Airborne fungal concentration at each sampling location.

Sampling sites		First week (CFU/m^3)	Second week (CFU/m^3)	Third week (CFU/m^3)
**BJD**	**C**	80	40	90
	**U**	40	90	180
**NG**	**C**	70	80	120
	**U**	30	60	100
**WJY**	**C**	10	120	60
	**U**	60	130	120
**WJR**	**C**	50	40	90
	**U**	10	120	50
**NMWA**	**C**	10	150	110
	**U**	50	60	60

Supplementary Table S5. Fungal genera distribution at U and C sites.

**Fungal genera**	**Total Strains count At C sites**	**Total Strains count At U sites**
Alternaria	23	24
Amphobotrys	0	1
Arthrinium	0	1
Aspergillus	4	5
Aureobasidium	0	4
Bipolaris	1	1
Botryosporium	1	0
Bullera	0	1
Cercospora	2	2
Chaetomium	1	0
Choanephora	1	0
Cladosporium	13	14
Clonostachys	0	1
Cochliobolus	0	1
Colletotrichum	1	1
Coprinellus	3	5
Coprinopsis	1	0
Curvularia	0	1
Deniquelata	1	0
Didymella	1	1
Epicoccum	0	1
Fusarium	3	1
Hirsutella	0	1
Hannaella	4	1
Irpex	2	1
Keissleriella	2	1
Lophiostoma	1	0
Magnaporthe	1	1
Moesziomyces	4	0
Neosetophoma	1	1
Nigrospora	1	0
Papiliotrema	1	1
Penicillium	4	5
Phaeosphaeria	0	1
Phoma	2	0
Psathyrella	0	1
Pseudopithomyces	0	1
Pseudozyma	0	1
Pyricularia	0	3

Rhizopus	0	1
Rhodosporidiobolus	0	1
Rhodotorula	0	1
Schizophyllum	7	7
Scopulariopsis	0	1
Septoriella	1	0
Setomelanomma	1	0
Sporidiobolus	4	7
Sporobolomyces	7	6
Torula	1	0
Trichoderma	5	0
Trichothecium	1	0
Typhula	1	0
**58 genera in total**	**112**	**116**

Supplementary Table S6: Environmental factors recorded in each sampling site at the time of sampling (C = center of the bridge; U = under the bridge).

Location	1st week		2nd week		3rd week	
	**Temperature (°C)**	**RH (%)**	**Temperature (°C)**	**RH (%)**	**Temperature (°C)**	**RH (%)**
Northgate (C)	25.3	79.9	22.9	93.1	29.6	77.2
Northgate (U)	24.2	79.0	23.0	93.2	29.6	77.2
Nanmenwai avenue (C)	23.2	63.4	27.9	93.2	28.6	77.2
Nanmenwai avenue (U)	23.6	63.2	27.5	93.2	28.6	77.2
Wujiayao avenue (C)	29.9	81.6	26.9	93.2	22.4	75.2
Wujiayao avenue (U)	30.0	81.6	25.9	93.2	21.8	74.3
Binjiangdao (C)	25.9	70.6	28.7	93.3	25.5	77.2
Binjiangdao (U)	26.1	70.6	28.0	93.3	24.2	77.2
Weijin road (C)	27.2	79.9	25.7	93.2	23.2	63.9
Weijin road (U)	27.0	79.9	23.3	93.2	23.1	74.1

a

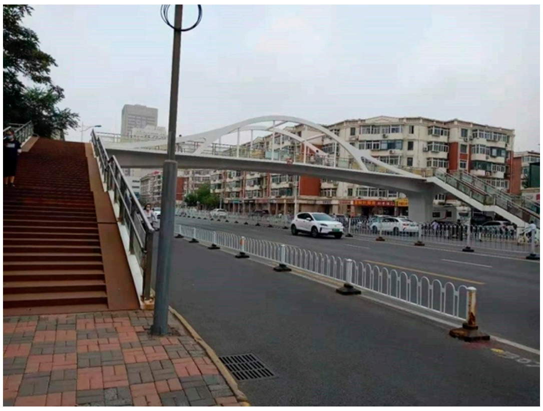

b

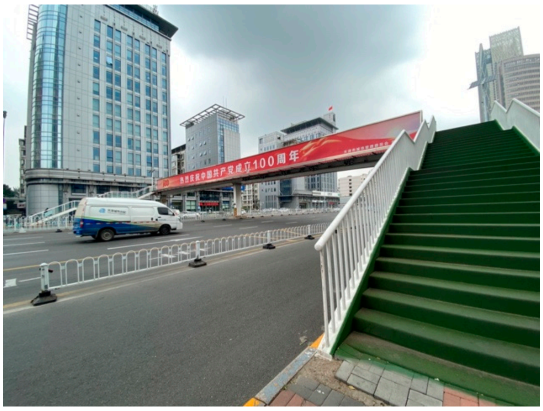

c

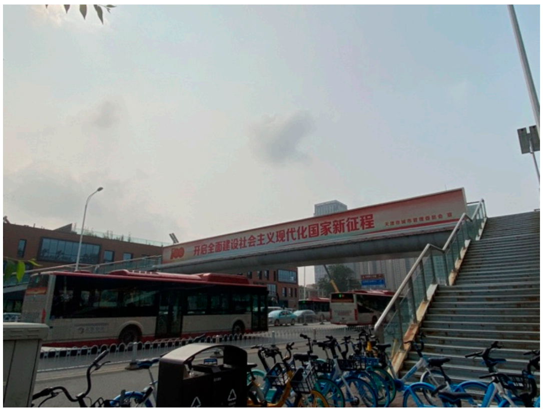

d

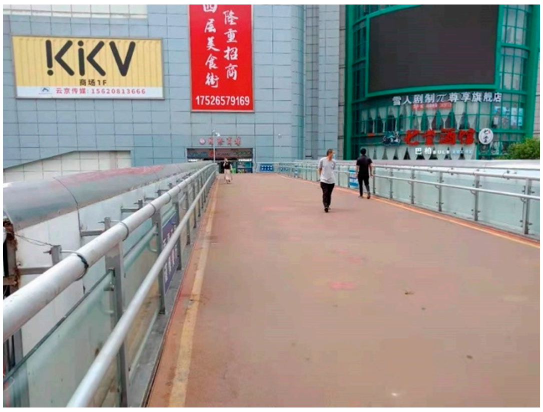

Supplementary Figure S1: Sampling sites. NG (a), NMWA (b), and WJR pedestrian bridges; top central area of BJD pedestrian bridge (d).

a b c d

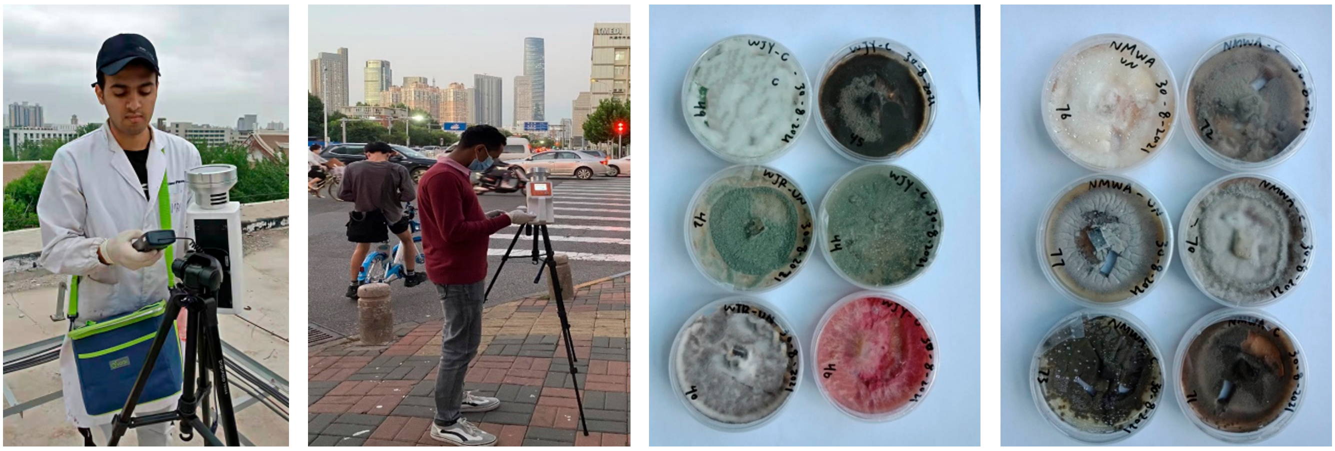

Supplementary Figure S2: Collection of air samples (a, b) and examples of airborne fungal strains isolated from the analyzed pedestrian bridges (c, d).

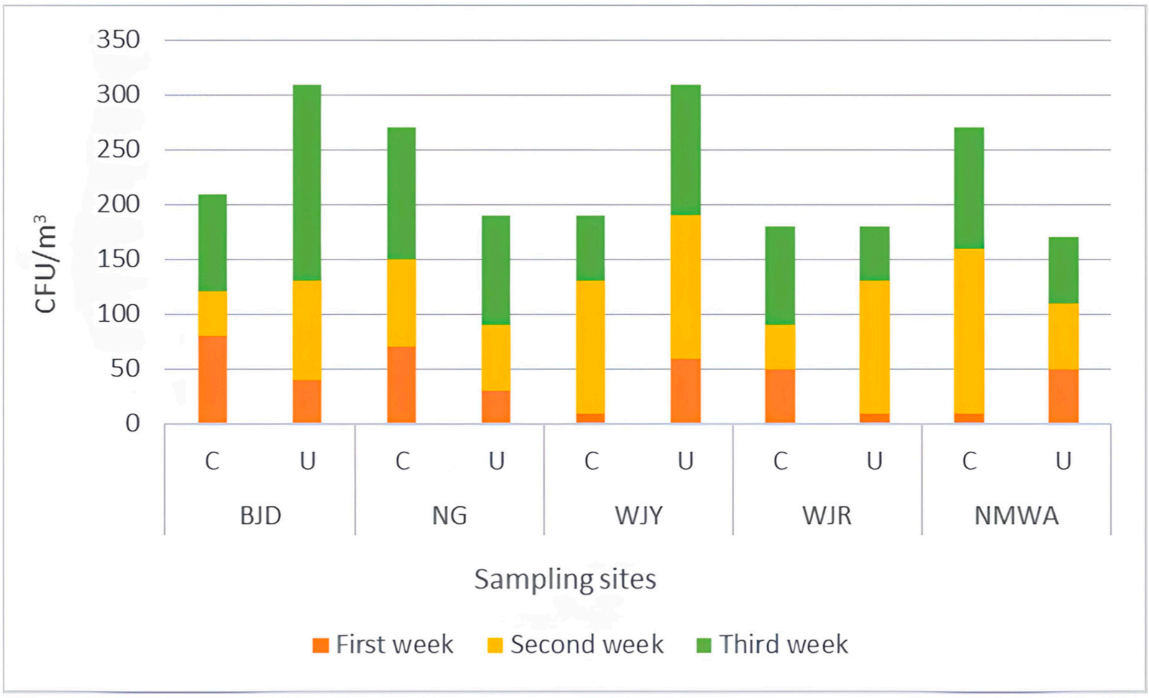

Supplementary Figure S3: Fungal concentration at each experimental location (C = the top central area of the bridges; U = under the bridges along the side walk).

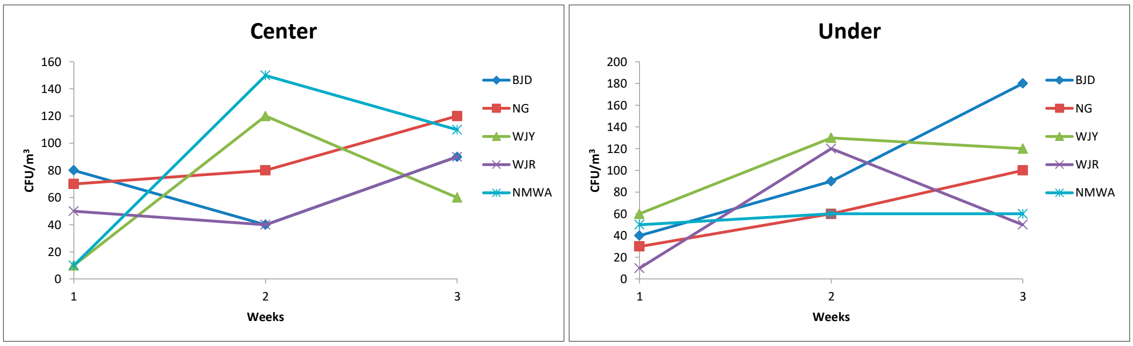

Supplementary Figure S4: Concentration of airborne fungi at the center and under the 5 bridges during the 3 weeks sampling period (CFU/m^3).

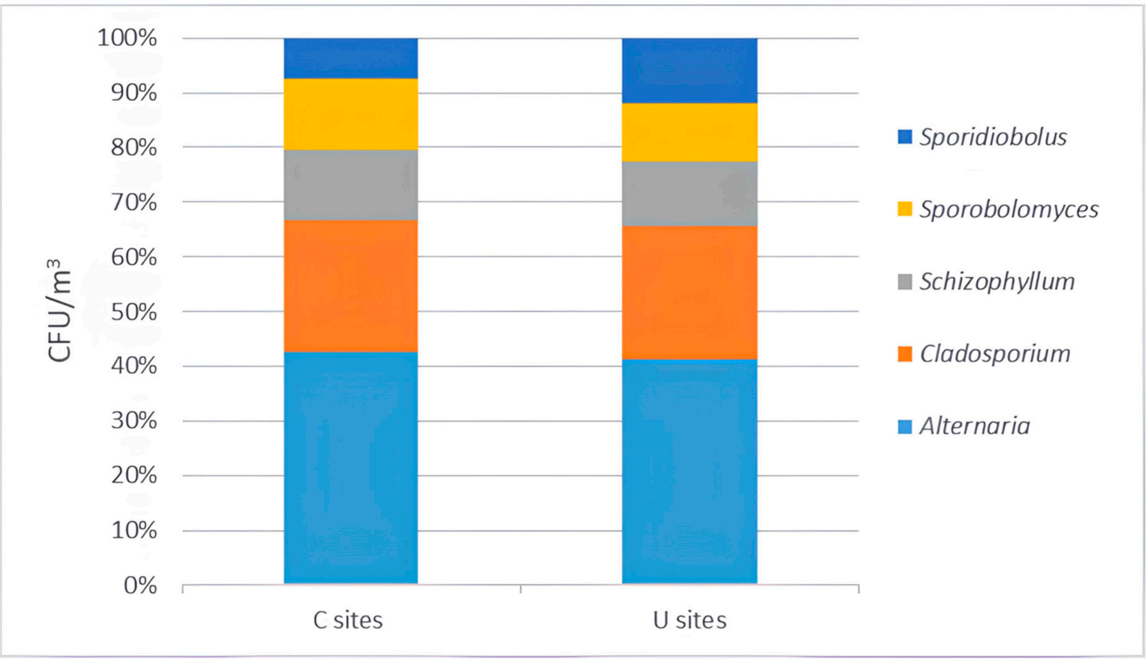

Supplementary Figure S5: Concentration of the five most abundant fungal genera collected from the two analyzed sites (C = the top central area of the bridges; U = under the bridges along the side walk) at each pedestrian bridge.

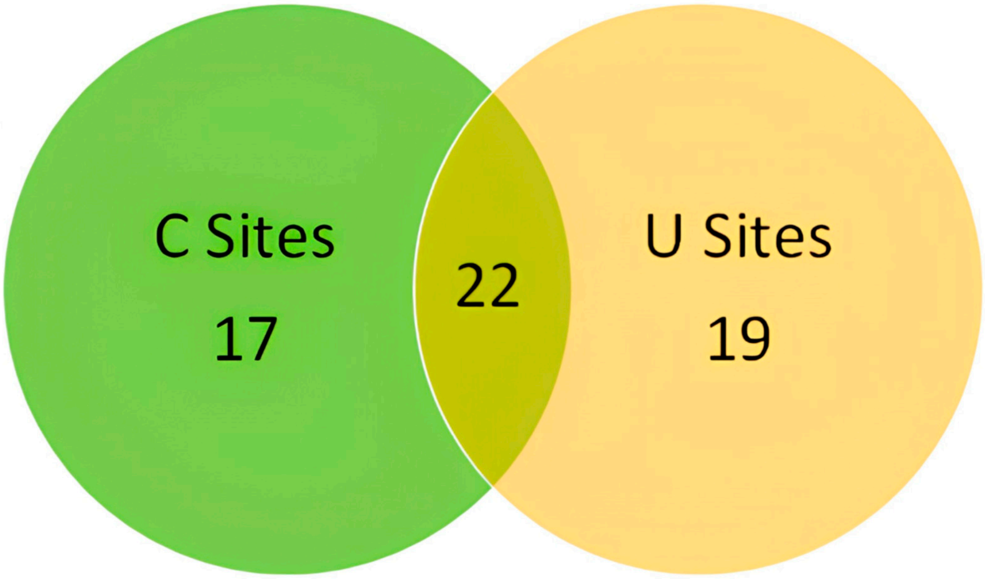

Supplementary Figure S6: Total number of fungal genera shared by both sites (C = the top central area of the bridges; U = under the bridges along the side walk) selected for each bridge.

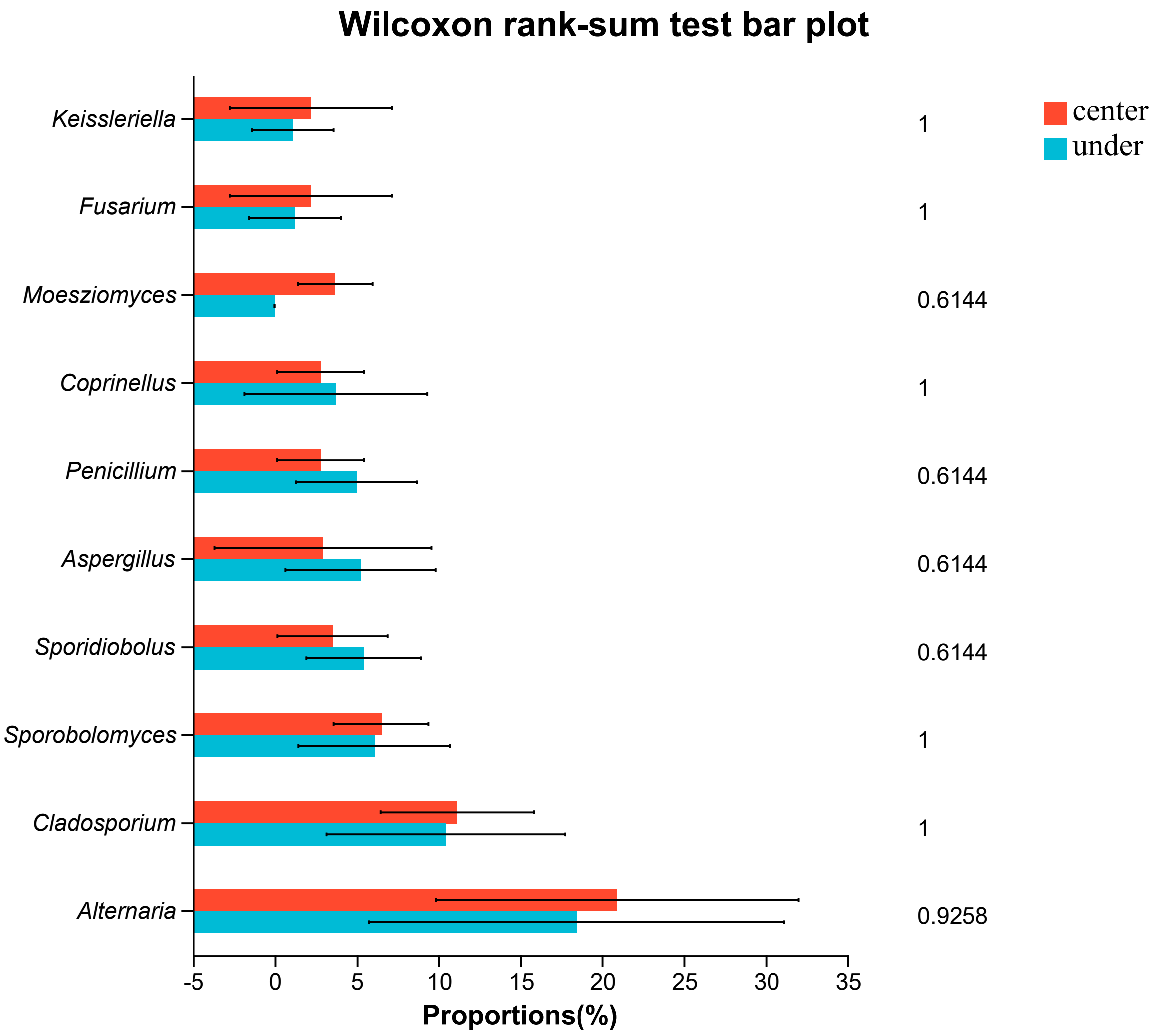

Supplementary Figure S7: Variation of ten most abundant fungal genera in the two sites at the center and under the investigated bridges, based on Wilcoxon rank-sum test. Error bars represent SEM.
